# A Critical Period for the Development of Schizophrenia-Like Pathology by Aberrant Postnatal Neurogenesis

**DOI:** 10.3389/fnins.2019.00635

**Published:** 2019-06-18

**Authors:** Joen-Rong Sheu, Cheng-Ying Hsieh, Thanasekaran Jayakumar, Mei-Fang Tseng, Hsing-Ni Lee, Shin-Wei Huang, Manjunath Manubolu, Chih-Hao Yang

**Affiliations:** ^1^Department of Pharmacology, School of Medicine, College of Medicine, Taipei Medical University, Taipei, Taiwan; ^2^Graduate Institute of Medical Sciences, College of Medicine, Taipei Medical University, Taipei, Taiwan; ^3^Department of Evolution, Ecology and Organismal Biology, The Ohio State University, Columbus, OH, United States

**Keywords:** aberrant adult neurogenesis, dendritic elaboration, maternal immune activation, proliferation, poly (I:C), schizophrenia

## Abstract

Schizophrenia is a complex and serious mental disorder, and patients with schizophrenia are characterized by psychological hallucinations, deregulated emotionality, and cognitive impairment. Evidence indicated that postnatal neurogenesis in the hippocampus is profoundly impaired in schizophrenic individuals but the role of such dysregulated neurodevelopmental processing in the pathophysiological progress of schizophrenia has not been well investigated. Here in this study, by using the rodent model of schizophrenia through maternal immune activation of poly (I:C) injection, we aimed to examine whether the postnatal neurogenesis might be involved in the development of schizophrenia-like pathology. Through the comprehensive behavioral analyses of multiple core symptoms of schizophrenia at different developmental stages (6-, 9-, and 12-weeks after birth) of the affected offspring, we found a delayed onset of schizophrenia-like behaviors in poly (I:C) animals through the development. Meanwhile, there is an age-dependent alteration of postnatal neurogenesis in the poly (I:C) animals along different development stages by which the aberrant dendritic elaboration functionally correlated with the schizophrenia-like symptoms in 9-week-old of age for the animals. Interestingly, increase in the neurogenesis during a critical period of neurodevelopment exacerbates the schizophrenia-like pathology. Conversely, temporal suppression of aberrant postnatal neurogenesis during the same period of neurodevelopment ameliorates the occurrence of schizophrenia-like symptoms. Together, these findings strongly suggested the aberrant dendritic growth of postnatal neurogenesis during the critical time window of development is essential for controlling the pathophysiological progression of schizophrenia-like symptoms. And pharmacological treatments that adjust these abnormalities may provide potential therapeutic benefits toward patients with schizophrenia in clinic.

## Introduction

Schizophrenia is a debilitating psychiatric disorder that affects approximately 1% of the general population worldwide (Perala et al., 2007; Global Burden of Disease Study 2013 Collaborators, 2015). The pathology of schizophrenia is characterized by positive symptoms (such as hallucinations, delusions, and thought disorder), negative symptoms (less emotional responsiveness and reduced motivations), and dysfunction of cognitive flexibility. Although acute psychosis can be controlled effectively with current antipsychotics, negative and cognitive symptoms are less responsive to such pharmacotherapy (Lieberman et al., 2005; Hanson et al., 2010; Ibrahim and Tamminga, 2012). Since the pathology of schizophrenia typically appears during the late adolescence or early adulthood, it has long been regarded as a neurodevelopmental disorder (Garey, 2010). In clinic, it has been found that most of the subjects diagnosed with schizophrenia has their first psychotic episode occurs in their late adolescence or early adulthood (Lewis and Lieberman, 2000; Lieberman, 2006).

Because of the delayed onset for the pathology of schizophrenia, considerable evidence suggests that the emergence of schizophrenia along the neurodevelopment might be linked to abnormal postnatal neurogenesis (Yun et al., 2016; Birnbaum and Weinberger, 2017). Neurogenesis, a process of giving birth to new neurons, was conventionally believed to take place only during embryonic development in mammals (Ramón y Cajal et al., 1991). Only recently it become generally accepted that neurogenesis is not restricted to prenatal stage but is a continuing process for the postnatal period, including the adolescence and even the adult. In the postnatal brain, the new neurons produced continuously at restricted brain regions including the subventricular zone and the subgranular zone of the hippocampal dentate gyrus throughout the whole life (Altman, 1969; Gage, 2002; Zhao et al., 2008).

Regarding the pathology of human schizophrenia, pathological features in the hippocampal neurogenic niche has been extensively investigated. Neuroimaging and postmortem studies have provided strong evidence showing the decreased hippocampal volume and functional connectivity between hippocampal subfields from the patients of schizophrenia (Heckers, 2001; Honea et al., 2005; Vita et al., 2006; van Erp et al., 2016). Meanwhile, histological analysis of postmortem tissue found a profoundly reduction in cell proliferation of the hippocampal dentate gyrus in schizophrenic adults (Barbeau et al., 1995; Reif et al., 2006). These previous studies have provided a causal link between the disruption of postnatal neurogenesis with the emergence of schizophrenia-like pathology along the neurodevelopmental process.

Meanwhile, accumulating epidemiological evidence suggested that prenatal infection or postnatal central nervous system infection with various pathogens such as viruses, bacteria, or parasites heighten the risk for several neuropsychiatric disorders including schizophrenia and autism spectrum disorders (Brown et al., 2004; Moreno et al., 2011). Based on the association, numerous animal models for schizophrenia have been established. One of the model is based on maternal exposure to the viral mimic: polyriboinosinic-polyribocytidilic acid (Poly I:C) in rodents. Poly (I:C) is a synthetic analog of double-stranded RNA which elicits cytokine-associated acute immune response (Kimura et al., 1994) and results in behavioral or structural abnormalities in the brain of the offspring that recapitulates the schizophrenia-like pathology in clinic (Zuckerman et al., 2003; Ozawa et al., 2006).

Although it has been postulated that the aberrant postnatal neurogenesis during the early brain development might be correlated to schizophrenia related psychosis, the question about whether the altered postnatal neurogenesis might be just a consequence of impaired brain maturation of schizophrenia or it might pathologically contribute to the emergence of schizophrenia related pathology along the brain development are still largely unanswered. Here in our current study, by using the rodent model of schizophrenia through maternal immune activation of poly (I:C) injection, we aimed to examine whether the postnatal neurogenesis might be directly involved in the development of schizophrenia-like pathology.

## Materials and Methods

### Animals

C57BL/6 mice of both sexes (BioLASCO, Taipei) were used for all the experiments including behavioral and histological experiments. Male and female C57BL/6 mice were bred and maintained by the Center for Laboratory Animal Research, at Taipei Medical University that maintained on a 12 h light/dark cycle (lights on at 7:00 am). All the animals had access *ad libitum* to food and water. All the experimental procedures for animal handling or surgeries were approved by the Institutional Animal Care and Use Committee (IACUC) of Taipei Medical University and in accordance with the guidelines for the Care and Use of Laboratory Animals provided by the National Institutes of Health.

### Maternal Immune Activation Model

For the mouse model of maternal immune activation in late gestation period, female mice were subjected for mating and checked daily at light cycle for the presence of seminal plugs as noted of gestation day 0.5 (GD0.5). On the gestation day 17 (GD17) pregnant dams were weighed carefully and received either a single injection of Poly (I:C) at a dose of 5 mg/kg or saline intravenously through the tail vein. Poly (I:C) which purchased from Sigma-Aldrich (St. Louis, MO, United States) was dissolved in sterile 0.9% saline solution to yield a final concentration of 1 mg/ml. After injection, the pregnant dam was returned to its home cage and left undisturbed until the delivery of its litter. All the litters were remained with the mother until the weaning of postnatal day 21 and then grouped housed as four mice with same-sex in a cage. In all the behavioral experiments, each experimental group was composed of no more than two mice that obtained from the same litter.

### Construction, Production, and Stereotaxic Injection of Engineered Retroviruses

High titers of engineered self-inactivating retroviruses (1 × 10^9^ unit/ml) expressing enhanced green fluorescent proteins were produced for the labeling and morphological analyses of newly generated neurons in the adult dentate gyrus. The expression of the GFP protein was constructed under the control of ubiquitin promoter (RV-Ubi-GFP) for a stable and reliable expression level in mature neurons. High titer retroviral particles were prepared by co-transfection of the RV-Ubi-GFP plasmid with the packaging vectors into the HEK-293 cells. And the medium that contained recombinant retroviruses was collected at 48 h after transfection for ultracentrifugation to concentrate the viral particles (Gu et al., 2012). For the labeling of newly generated neurons in the hippocampal dentate gyrus, the concentrated retroviruses (0.5 μl per site) were stereotaxically injected at four sites with the following coordinates: anterioposterior = −2 mm from bregma; mediolateral = ± 1.6 mm and dorsoventral = 2.5 mm; anterioposterior = −3 mm from bregma; mediolateral = ± 2.6 mm; and dorsoventral = 3.2 mm. And then the injection needle was kept in place for another 5 min after viral injection to avoid the back flow of the viral solution.

### Tissue Sectioning, Imaging, and Quantification

For the dendritic morphology analyses of retroviral labeling newly generated neurons, brain sections were prepared at 14 days after viral injection. Basically, mice were perfused transcardially with PBS followed by 4% formaldehyde under deep anesthesia by 5% isoflurane that mixed and maintained with 75% air and 25% of oxygen. After decapitation, the brain was removed and incubated overnight with 4% formaldehyde for fixation and then transferred in 30% sucrose solution at 4°C before sectioning. Sixty microliters coronal brain sections were prepared by the sliding Microtome (Leica, Nußloch, Germany) and the sections with the hippocampal dentate gyrus were used for the imaging of retroviral labeled newly generated neurons. The sectioned slices were washed with phosphate-buffered solution and incubated with 0.1 μg/ml of DAPI in PBS solution for 30 min to gain the counterstaining of granular cell layer from the hippocampal dentate gyrus. Slices were mounted and covered with the mounting solution (Vector Laboratories) on a glass coverslip and images of dendritic elaboration of individual GFP-expressing were imaged by the confocal spectral microscope imaging system (Leica TCS SP5) through the 40×, 1.40 NA oil immersion objective (Mannheim, Germany) by using argon or krypton laser. And then three-dimensional reconstructions of the complete dendritic elaboration from individual newly generated neurons were processed and projected. The morphological indexes such as length of primary dendrite, number of branches and total dendritic length of individual neurons were analyzed by the Neuron J plugin of the software ImageJ (NIH, United States).

### BrdU Labeling

To label the proliferating postnatal neurogenesis, Bromodeoxyuridine (BrdU) obtained from Sigma-Aldrich was injected intraperitoneally at a dose of 50 mg/kg to mark the dividing cells. BrdU was first dissolved in 0.1 M PBS and heated to 50–60°C to the concentration of 10 mg/ml. Each animal received two rounds of BrdU injection with the dosing interval of 12 h. Animals were sacrificed and brain sectioned for the immunohistochemical staining for the analyses of BrdU incorporated proliferating cells at 1 week after the BrdU injection.

### Immunohistochemistry and Stereological Analyses

Mice were perfused transcardially with PBS followed by 4% paraformaldehyde and post-fixed in PFA overnight then transferred to 30% sucrose solution. Coronal brain sections (40 μm) containing the hippocampi were prepared by the sliding Microtome (Leica, Germany). Immunohistochemical staining was performed on free-floating brain sections with every sixth brain section throughout the hippocampus (bregma: from −0.94 to −4.04 mm). For visualization of BrdU incorporation, DNA was denatured in 2N hydrochloric acid for 30 min at 37°C and then rinsed in 0.1 M borate buffer (pH 8.5) and washed extensively by 0.1 M PBS. And then brain sections were incubated overnight with primary antibody of rat anti-BrdU (1:2000, OBT0030, Bio-Rad) and goat anti-DCX (1:500, sc-8066, Santa Cruz, CA, United States) in PBS supplemented with 0.1% Triton X-100 and 3% donkey serum at 4°C. After extensive washes by PBS on the following day, the brain sections were incubated overnight with secondary antibodies as donkey Alexa Fluor 488 anti-rat (1:2000, A21208) and donkey Alexa Fluor 594 anti-goat (1:2000, A11058, Thermo Fisher Scientific) at 4°C. After three times of PBS washes, the sectioned slices were incubated with DAPI in PBS for 30 min. Slices were mounted and covered with the mounting solution (Vector Laboratories) on a glass coverslip and images from different florescent channels were obtained by the confocal spectral microscope imaging system (Leica TCS SP5) through the 40×, 1.40 NA oil immersion objective (Mannheim, Germany).

For the stereological analyses of brain sections, confocal stacked images of areas containing newly generated neurons in the dentate gyrus were first obtained for all the immunohistochemical stained brain slices (every sixth brain section throughout the hippocampus). BrdU-positive cells were counted from individual confocal *Z*-stack images (every six brain sections across the whole hippocampus) and averaged to obtain the BrdU-positive cells per brain section for individual animal. And DCX-positive cells were counted from individual confocal *Z*-stack images and calculated as the DCX-positive immature neurons per brain section. Meanwhile, the percentage of BrdU positive in DCX positive cells was calculated by the confocal *Z*-stack images to analyze the percentage of BrdU positive cells that co-express the immature neuronal marker of DCX.

### Behavioral Analyses

For the behavioral analyses of different core schizophrenia-like symptoms, C57BL/6 mice of both sexes were used for all the behavioral experiments in this study. And equal amounts of male or females were tested in different behavioral experiments to exclude the gender bias for the data analyses. Two to three different behavioral analyses were designed for the same animals at their indicted developmental period of age to reduce the total number of animals required for the whole study and animals were allowed to rest for at least 24 h between two behavioral tests. Meanwhile, no of the animals were conducted with the same behavioral test more than once to avoid the habituation of the repeated behavioral analyses.

### Novelty Suppressed Feeding Test

The testing chamber is composed of a white-color rectangular box (40 cm × 40 cm × 40 cm) with a camera mounted on the top of the chamber for recording of the animals’ feeding behavior. And the floor of the testing chamber was covered with fresh bedding for each testing mouse. A standard food pellet (around 20 g) was placed at the center of the box. After fasting (food-deprived) for 24 h, the testing mouse was placed individually at the corner of the box that facing the center food pellet. And the latency for the mouse to start biting or chewing the pellet was measured for a maximum testing duration of 10 min. And then the mouse was returned to its home cage with a weighed food pellet (around 20 g), and the latency for the mouse to feed and total amount consumed in 5 min were analyzed to evaluate the non-specific changes in the motivation of feeding for the testing mouse.

### MK-801 Induced Hyperlocomotion

The experimental apparatus consisted of a white-color rectangular box (40 cm × 40 cm × 40 cm) with the video camera mounted on the top of the box for recording of the animals’ locomotion activity. For the evaluation of basal locomotor activity, mice were allowed to freely explore the box for 60 min. After the habituation to the testing chamber, the mouse was intraperitoneally injected with MK-801 and monitored for the MK-801 induced locomotor changes for an additional 90 min. MK-801 which purchased from Sigma-Aldrich (+)-MK-801 hydrogen maleate, M107) was first dissolved in sterile 0.9% saline solution to yield a stock solution of 1 mg/ml. Two doses of MK-801 (0.2 and 0.4 mg/kg) induced hyperlocomotion were tested in our current study but only the results of 0.4 mg/kg MK-801 injection were presented to simplify the presentation of the figures. All locomotor experiments were carried out under a dimmed light condition (around 25 lux). The locomotor behavior of the mouse was video-recorded and the total distance traveled was automatically analyzed by the behavioral tracking system Ethovision XT (Noldus).

### Object Location Memory Task

The object location memory task was used to evaluate the hippocampal-dependent spatial recognition memory. The experimental apparatus consisted of a white-color rectangular box (40 cm × 40 cm × 40 cm) with a camera mounted on the top of the chamber for recording of the animals’ exploratory behavior. Multiple visual cues were attached on the walls of the testing apparatus to provide contextual guiding information. The procedure was divided into three sections: habituation, training and testing phases. For the 10-min habitation phase, the animals were exposed for exploration the box without any testing objects to acclimatize the animals to the testing apparatus. During the training phase, the testing mouse was placed in the arena for exploration of two identical objects located at two different quadrants of the apparatus for 10 min and returned to their home cage. After a period of 30-min delay, the testing mouse was returned to the testing apparatus in which one of the original objects had been repositioned to another corner of the chamber. Objects and their placement in the testing chamber were varied counterbalanced between groups to avoid positional biases. The testing phase lasted for 5 min and the time duration for the mouse to explore the two objects was video-recorded and analyzed by the behavioral tracking system Ethovision XT (Noldus). The discrimination index which reflects the cognitive performance of individual preference to the repositioned object was calculated by the formula of [time spent on the object in novel location/(time spent on the object in novel location + time spent on the object in familiar location)] × 100%.

### Open Field Test

The thigmotactic behavior performed in an open arena was evaluated for the mice to reflect the anxiety-like behaviors. Briefly, the testing animal was placed individually at the center of a rectangular testing chamber (40 cm × 40 cm × 40 cm) with the video camera mounted on the top of the box for recording of the animals’ exploratory behavior. A lamp of dimmed light source with 25 lux was illuminated from the overhead and the animals were allowed to freely explore the arena for 10 min. The central zone (25% of the center) was defined as the 20 cm × 20 cm at center portion of the box. The number of entry and the percentage of time spent in the central zone for the mouse was analyzed behavioral tracking system Ethovision (Noldus). The box was cleaned with 75% ethanol between trials to avoid interference of the analyses between different testing animals.

### Sucrose Preference Test

The testing mice were first habituated to a two-bottle-choice paradigm for consuming the water for 3–5 days. And then on the testing day, the experimental mouse was housed individually in the testing chamber which is identical to its home cage. And then, it was allowed to choose the solution intake from either the bottle of tap water or the other bottle with 3% sucrose solution. Consumption of the either solution was measured 24 h later for the calculation of sucrose preference ratio: the volume of sucrose consumed/the total liquid consumption × 100%.

### Tail Suspension Test

Animals were transferred to the experimental room and allowed to acclimate to the environment for at least 3 h on the day of testing. The mice were individually suspended by the tail from a horizontal bar (distance from floor = 35 cm) by using adhesive tape wrapped around one cm from tip of the tail. A 5 min testing trial was applied and the escape-oriented behaviors and bouts of immobility for the animals was video recorded by a camera positioned in front of the testing apparatus. And the total duration of immobility for individual mouse was analyzed by experienced observer. Because certain number (10–20%) of C57BL/6 mice might climb up their tails during the testing session, mice that climbed their tail were excluded from the final analyses.

### Spontaneous Alternation Y Maze Test

The Y maze test was applied to assess the spatial working memory performance for the mice. The testing apparatus is composed of three identical acrylic white-colored arms with equal angles between each arm measuring 25 cm long, 6 cm wide, and 20 cm high. Mice was placed individually at the end of a randomly selected arm as a starting point for the test and allowed to freely explore the Y maze for 10 min. And the sequential order of each arm visits was video-recorded and analyzed for the behavioral spontaneous alternation. Each arm visit was defined as the mouse moving of all four paws into the specific arm and the alteration was defined as the consecutive entry into three different arms. The percentage of alteration (%) was calculated by the formula: total number of alternations/maximum possible alternations (total number of arms entered-2) × 100.

### Pharmacological Stimulation or Suppression of Neurogenesis

For the experiments to increase in postnatal neurogenesis, memantine (MEM) at a dose of 10 mg/kg which has been found to enhance the proliferation of radial glia-like progenitor cells (Sun et al., 2015) was applied to increase the neurogenesis in hippocampal dentate gyrus. Memantine which purchased from Sigma (M9292) was dissolved in sterile 0.9% saline solution to yield a final concentration of 1 mg/ml. And then the animals received memantine treatment (10 mg/kg) through intraperitoneal injection every 2 days for a period of 2 weeks before behavioral analyses. For the experiments to temporal suppress postnatal neurogenesis, temozolomide (TMZ) was used to block the active cell division of neural stem cells. Temozolomide (T2577) which purchased from Sigma was dissolved in sterile 0.9% saline solution to yield a final concentration of 2.5 mg/ml. The animals with temozolomide treatment received a procedure of 2-week intraperitoneal injections of TMZ which composed of 25 mg/kg TMZ injection once a day for three consecutive days followed by 4 days of non-injection recovery. Such injection regimen was adapted from the glioma treatment in clinic and has been shown to reduce neurogenesis in mice effectively (Garthe et al., 2009). Two cycles of TMZ injection was applied for the TMZ treatment group of animals, whereas the vehicle group received injection of saline solution only.

### Genetic Suppression of Postnatal Neurogenesis

Nestin-CreERT2 mice were obtained from the Jackson Laboratory and maintained on a C57BL/6 background. High titers of AAV-flex-DTA viruses (1 × 10^12^ unit/ml) were produced by HEK-293 cells and concentrated by AAVanced concentration reagent (System Biosciences). The concentrated AAV-flex-DTA viruses were stereotaxically injected into the dentate gyrus of Nestin-CreERT2 mice at 5-week old of the animals. And then the animals received daily injection of 150 mg/kg tamoxifen (in corn oil; Sigma) intraperitoneally for five consecutive days started from 7-week of age for the mouse to induce the expression of diphtheria toxin in Nestin positive cells.

### Statistical Analysis

All the results are presented as means ± SEM. To demonstrate the interaction of different independent variables for the behavioral or morphological experiments in the study, the analyses for significant difference between multiple groups were first analyzed with two-way analyses of variance (ANOVAs) followed by Bonferroni’s *post hoc* analyses for evaluating the significance between isolated experimental groups. And Pearson’s correlation analysis was performed for the study of correlation analyses between two parameters. Probability values of *P* < 0.05 were considered to represent statistical significance. All the statistical analyses were performed using the software of Prism 6 (GraphPad).

## Results

### Delayed Emergence of Schizophrenia-Like Symptoms in the Poly (I:C) Animals Along the Development

**FIGURE 1:**
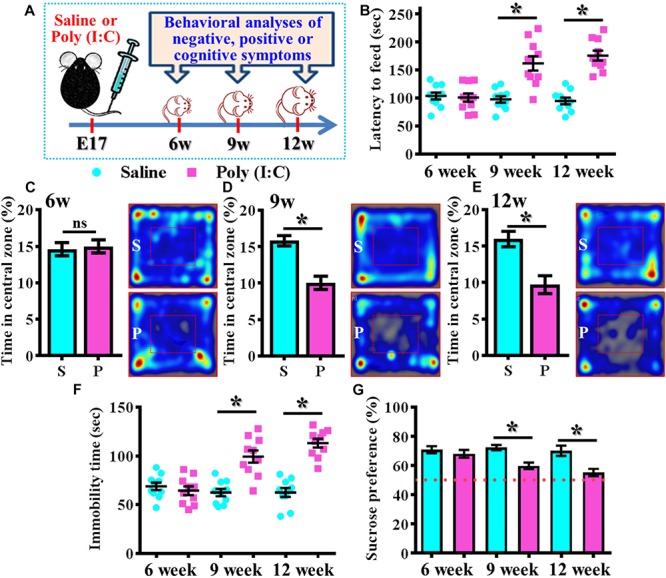
Development of anxiety- and depression-like symptoms in the poly (I:C) animals along the developmental stages. **(A)** Schematic illustration of the procedure of experimental designs. **(B)** Scatter plot showing the behavioral performance of novelty suppressed feeding at different developmental stages. 2-way ANOVA: Age (6, 9, 12): *F*(2,54) = 9.191, *P* = 0.0004; treatment (saline vs. poly I:C): *F*(1,54) = 49.76, *P* < 0.0001; interaction: *F*(2,54) = 14.52, *P* < 0.0001 and analyzed with Bonferroni’s *post hoc* analysis. **(C–E)** Quantification and representative images of percentage in the central open zone at **(C)** 6-, **(D)** 9-, and **(E)** 12-week of age. Two-way ANOVA: Age (6, 9, 12): *F*(2,54) = 2.644, *P* = 0.0803; treatment (saline vs. poly I:C): *F*(1,54) = 24.55, *P* < 0.0001; interaction: *F*(2,54) = 7.427, *P* = 0.0014. S or P in the figures of heatmap indicate saline or poly (I:C) animals, respectively. **(F)** Scatter plot showing the behavioral performance of tail suspension test. Two-way ANOVA: Age (6, 9, 12): *F*(2,56) = 11.25, *P* < 0.0001; treatment (saline vs. poly I:C): *F*(1,56) = 54.84, *P* < 0.0001; interaction: *F*(2,56) = 19.49, *P* < 0.0001. **(G)** Quantification of preference to 3% sucrose solution in the two-bottle preference test. Two-way ANOVA: Age (6, 9, 12): *F*(2,58) = 4.081, *P* = 0.0220; treatment (saline vs. poly I:C): *F*(1,58) = 25.93, *P* < 0.0001; interaction: *F*(2,58) = 3.511, *P* = 0.0364. Data are presented as mean ± SEM. *n* = 10–12 mice. ^*^P < 0.05. “ns” presents no significant.

By using the rodent model of schizophrenia through maternal immune activation with poly (I:C), we first confirmed the presence of core symptoms (negative-, positive-symptoms or cognitive impairment) of schizophrenia-like pathology at different neurodevelopmental stages of the animals. Experimentally, the offspring of the saline or poly (I:C) injected dams were tested with a battery of behavioral analyses to reflect the core symptoms of schizophrenia-like pathology at 6-, 9-, and 12-week of age (Figure 1A). By using the model of novelty-suppressed feeding, we found the poly (I:C) group of animals displayed a prolonged latency for start eating in the novel context. ANOVA analyses indicated that there is an age-dependent difference between the animals, and there is a significant increase in the latency for feeding in poly (I:C) group of animals (Figure 1B, *P* < 0.05 at 9 and 12 week of age vs. saline groups). Meanwhile, a thigmotactic behavior performed in an open arena was also conducted for the mouse. We found there is a significant reduction in the duration of central open zone entry for the poly (I:C) group of animals in an age-dependent manner. Compared to saline groups of animal, a significant decrease of the time in central zone was evident in 9- (Figure 1D, *P* < 0.05) and 12-week of age (Figure 1E, *P* < 0.05) of the poly (I:C) group of animals but not in the age of 6-week-old (Figure 1C). Both of the behavioral alterations are indication of the increase in anxiety-like symptoms for the animals.

Besides, the behavioral despair of the animals was examined by the tail suspension test at different developmental stages of the offspring. We found there is a significant increase in the immobility time for the poly (I:C) group of animals (Figure 1F, *P* < 0.05 at 9 and 12 week of age vs. saline groups). But no difference was observed for the animals at their 6-week-old of age. Meanwhile, the poly (I:C) group of animals also displayed an age-dependent increase in the symptoms of anhedonia when tested with the two-bottle sucrose preference test (Figure 1G). Compared to saline group, there is a significant reduction of the hedonic drive for sucrose solution from poly (I:C) group of animals at their 9 and 12-week of age (Figure 1G, *P* < 0.05). Both of the behavioral changes reflected an increase in depression-like symptoms of the animals.

**FIGURE 2:**
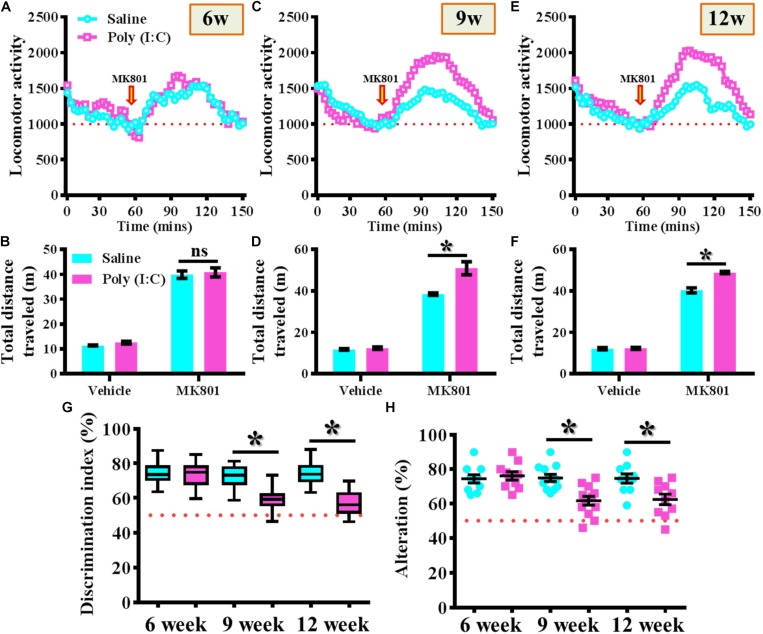
Maternal immune activation altered the MK-801-induced locomotor changes and cognitive performance along different developmental stages. Basal locomotor activity and MK-801-induced hyperlocomotion at **(A)** 6-, **(C)** 9-, and **(E)** 12-week of age. Data are presented with the average value from ten mice and each data point represents the cumulative distance traveled for a time bin of 3 min. Arrow indicated the time point of MK-801 injection. Quantification of basal locomotor activity (vehicle injected) and MK-801-induced hyperlocomotion at **(B)** 6-, **(D)** 9-, and **(F)** 12-week of age. Data are presented as mean ± SEM. *n* = 10 mice, data are analyzed by two-way ANOVA between animals from **(B,D,F)** and further analyzed with Bonferroni’s *post hoc* analysis. ^*^P < 0.05 compared to saline group of animals. “ns” presents no significant. **(G)** Box-and-whisker plots showing the cognitive performance of object location memory task. Two-way ANOVA: Age (6, 9, 12): *F*(2,54) = 8.756, *P* = 0.0005; treatment (saline vs. poly I:C): *F*(1,54) = 31.33, *P* < 0.0001; interaction: *F*(2,54) = 6.414, *P* = 0.0032. **(H)** Scatter plot showing the percentage of spontaneous alternation in the Y maze test. Two-way ANOVA: Age (6, 9, 12): *F*(2,58) = 4.75, *P* = 0.0123; treatment (saline vs. poly I:C): *F*(1,58) = 14.59, *P* = 0.0003; interaction: *F*(2,58) = 5.298, *P* = 0.0077. All the data are presented as mean ± SEM. *n* = 10–12 mice. ^*^P < 0.05.

MK-801 (also known as Dizocilpine) induced hyperlocomotion has long been used as the rodent model for reflecting the positive-like psychotic symptoms of schizophrenia. Experimentally, the offspring of the saline or poly (I:C) injected dams were tested for their basal locomotor activity or MK-801-induced hyperlocomotion at 6-, 9-, and 12-week of age (Figures 2A–F). We found there is no significant difference in the basal locomotor activity between the saline or poly (I:C) group of animals (the vehicle injected experiment in Figures 2B,D,E). Interestingly, there is a significant increase in MK-801 (0.4 mg/kg) induced locomotor changes for the poly (I:C) group of animals at 9 and 12-week of age (Figures 2C–F, *P* < 0.05) but not at the age of 6-week-old (Figures 2A,B).

Besides, the object location memory task and spontaneous Y maze alternation test were used for the evaluation of cognitive performance or spatial working memory changes for the animals at different developmental stages of the offspring. By using the object location memory task, we found an age-dependent loss in hippocampus dependent memory function for the poly (I:C) group of animals at 9 and 12-week of age (Figure 2G, *P* < 0.05) but not in the age of 6-week-old (Figure 2G). Meanwhile, the spatial working memory performance evaluated by calculating the spontaneous alternation in the Y maze indicated that there is a significant reduction in the percentage of alternation in arm choice for the poly (I:C) group of animals in an age-dependent manner. Compared to saline groups of animal, a significant reduction in alteration of arm choice was found in 9- and 12-week of age (Figure 2H, *P* < 0.05) of the poly (I:C) group of animals but not at their age of 6-week-old (Figure 2H, 6-week-old saline, 74.4 ± 2.418% vs. 6-week-old poly (I:C), 76.1 ± 2.406%).

Through the rodent model of maternal immune activation, we first confirmed the gradual occurrence of schizophrenia-like pathology along the developmental stage of the animals. And these behavioral observations reflect the core symptoms of schizophrenia indicated our poly (I:C) model, which could recapitulate the neurodevelopmental nature of schizophrenic pathology in human patients.

### Altered Postnatal Neurogenesis in the Poly (I:C) Animals Along the Neurodevelopment

**FIGURE 3:**
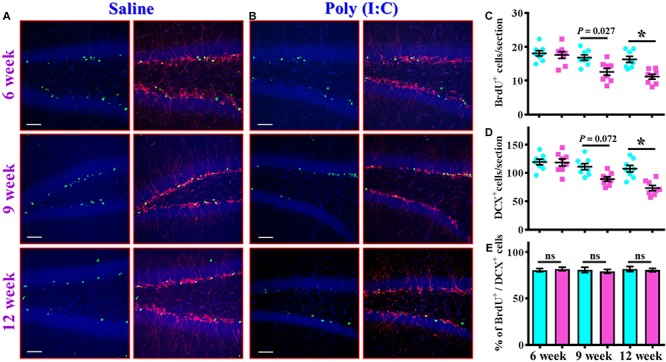
Altered postnatal neurogenesis in the poly (I:C) animals along the neurodevelopment. **(A,B)** Representative images for the double staining of BrdU and DCX from the saline **(A)** or poly (I:C) animals **(B)** at different developmental stages. Green, BrdU^+^ cells. Red, doublecortin (DCX)^+^ cells. Blue, DAPI signal. The scale bar is 50 μm. **(C)** Scatter plots showing the number of BrdU positive cells per hippocampal section at different developmental stages. Two-way ANOVA: Age (6, 9, 12): *F*(2,42) = 11.59, *P* < 0.0001; treatment (saline vs. poly I:C): *F*(1,42) = 19.99, *P* < 0.0001; interaction: *F*(2,42) = 3.734, *P* = 0.0322 and analyzed with Bonferroni’s *post hoc* analysis. **(D)** Scatter plots showing the number of DCX positive cells per hippocampal section at different developmental stages. Two-way ANOVA: Age (6, 9, 12): *F*(2,42) = 15.21, *P* < 0.0001; treatment (saline vs. poly I:C): *F*(1,42) = 19.79, *P* < 0.0001; interaction: *F*(2,42) = 5.174, *P* = 0.0098. **(E)** Quantification of the percentage for BrdU positive cells that co-expressed the protein doublecortin at different developmental stages. Two-way ANOVA: Age (6, 9, 12): *F*(2,42) = 0.1812, *P* = 0.8349; treatment (saline vs. poly I:C): *F*(1,42) = 0.086, *P* = 0.7708; interaction: *F*(2,42) = 0.2467, *P* = 0.7825. Data are presented as mean ± SEM, *n* = 8 mice, ^*^P < 0.05. “ns” presents no significant.

The findings of delayed emergence of schizophrenia-like symptoms along the neurodevelopmental process raised the possibility of aberrant postnatal neurogenesis that involved in its pathology. We first evaluated the number of proliferating cells in the neurogenic niche of the hippocampal dentate gyrus from the offspring of the saline or poly (I:C) injected dams at 6-, 9-, and 12-week of age (Figures 3A,B). By analyzing the number of BrdU incorporated cells in the SGZ of the hippocampus, we found there is a gradual decrease in the proliferating capacity of newborn cells from the poly (I:C) group of animals along the development of the animals. This was evidenced by a significant reduction in the number of BrdU positive cells in the poly (I:C) group of animals at both 9- and 12-week of age [Figure 3C, 9-week-old Saline, 16.8 ± 0.8075 cells vs. 9-week-old poly (I:C), 12.59 ± 1.047 cells, *P* < 0.05; 12-week-old Saline, 16.26 ± 0.8919 cells vs. 12-week-old poly (I:C), 11.17 ± 0.7345 cells, *P* < 0.05].

Similar results were also confirmed by the staining of immature neuronal marker of doublecortin protein, and we found there is a gradual decrease in the DCX positive cells in the poly (I:C) animals at their 12-week of age vs. saline group (Figure 3D, *P* < 0.05). Meanwhile, we also analyzed the percentage of BrdU positive cells that co-express the immature neuronal marker of DCX between the offspring of the saline or poly (I:C) injected dams at different developmental stages. Our data indicated that there was no significant difference in the percentage of BrdU^+^/DCX^+^ cells between saline or poly (I:C) group of animals for all the three developmental stages tested (Figure 3E). Together, these observations indicated that maternal immune activation might induce a negative influence on the proliferation of postnatal neurogenesis.

**FIGURE 4:**
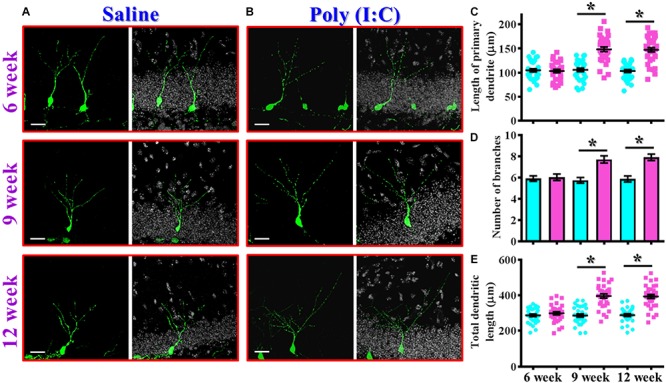
Aberrant dendritic development of the newly generated neurons in the poly (I:C) animals. **(A,B)** Representative images for the retroviral labeled newly generated neurons at 14 days post-retroviral injection from the saline **(A)** or poly (I:C) **(B)** animals at different developmental stages. Green, EGFP signal from labeled cells. White, DAPI signal. The scale bar is 25 μm. **(C)** Scatter plots showing the length of primary dendrite of individual newborn neurons at different developmental stages. Two-way ANOVA: Age (6, 9, 12): *F*(2,189) = 22.33, *P* < 0.0001; treatment (saline vs. poly I:C): *F*(1,189) = 83.11, *P* < 0.0001; interaction: *F*(2,189) = 22.72, *P* < 0.0001 and analyzed with Bonferroni’s *post hoc* analysis. **(D)** Quantification of the number of dendritic branches from the newly generated neurons at different developmental stages. Two-way ANOVA: Age (6, 9, 12): *F*(2,189) = 5.693, *P* = 0.004; treatment (saline vs. poly I:C): *F*(1,189) = 33.18, *P* < 0.0001; interaction: *F*(2,189) = 6.824, *P* = 0.0014. **(E)** Scatter plots showing the total dendritic length of individual newborn neurons at different developmental stages. Two-way ANOVA: Age (6, 9, 12): *F*(2,189) = 18.06, *P* < 0.0001; treatment (saline vs. poly I:C): *F*(1,189) = 100.6, *P* < 0.0001; interaction: *F*(2,189) = 17.94, *P* < 0.0001. Data are presented as mean ± SEM in **(C–E)**. *n* = 30–40 cells obtained from six mice. ^*^P < 0.05.

In addition to proliferation and neuronal differentiation, the postnatal newly generated neurons might elaborate the dendrites for their proper integration into the existing circuits. We then investigated if the dendritic development of the newly generated neurons might be affected in the rodent model of schizophrenia. Retroviral labeling of newly generating neurons was analyzed at 14 days after retroviral injection for the visualization of dendritic maturation of postnatal neurogenesis (Figures 4A,B). We found there is an aberrant dendritic development of the newly generated neurons from the poly (I:C) group of animals in an age-dependent manner. Quantitative analyses indicated a significant increase in the length of primary dendrite (Figure 4C, *P* < 0.05), the number of dendritic branches (Figure 4D, *P* < 0.05) and the total dendritic length (Figure 4E, *P* < 0.05) of the newly generated neurons from the poly (I:C) group of animals at 9 and 12-week of age but not at their age of 6-week-old.

Together, these observations indicated an aberrant postnatal neurogenesis in our rodent model of schizophrenia. Meanwhile, the delayed onset of changes in morphological phenotypes along the neurodevelopmental process correlated with the time course of emergence in schizophrenia-like symptoms which strongly suggested a possible role for aberrant neurogenesis in the pathology of schizophrenia.

### Aberrant Dendritic Development Functionally Correlated With the Schizophrenia-Like Symptoms

**FIGURE 5:**
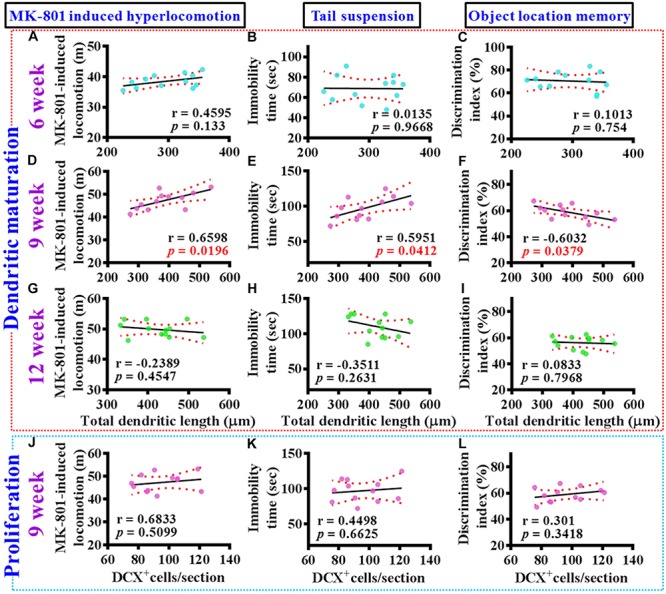
Aberrant dendritic elaboration functionally correlated with the schizophrenia-like symptoms. **(A–C)** Pearson’s correlation plots between the total dendritic length obtained from the averaged value of 8–10 labeled cells by individual poly (I:C) animals with their different behavioral indexes at 6-week of age. *n* = 12 mice. **(D–F)** Pearson’s correlation plots between the total dendritic length averaged from 8 to 10 labeled cells by individual poly (I:C) animals with their different behavioral indexes at 9-week of age. *n* = 12 mice. **(G–I)** Pearson’s correlation between the total dendritic length averaged from 8 to 10 labeled cells by individual poly (I:C) animals with their different behavioral indexes at 12-week of age. *n* = 12 mice. **(J–L)** Pearson’s correlation plots showing the number of DCX positive cells averaged from five hippocampal sections by individual poly (I:C) animals with their different behavioral indexes at 9-week of age. *n* = 12 mice.

To gain more insight of the altered postnatal neurogenesis in the pathology of schizophrenia, we next conducted the correlation analyses of altered neurogenesis with variety of behavioral indexes from individual poly (I:C) group of animals at 6-, 9-, and 12-week of age. No significance in the correlation of total dendritic length to different behaviors was found for the poly (I:C) animals at 6-week of age (Figures 5A–C). Interestingly, a significant correlation of total dendritic length to MK-801-induced hyperlocomotion (Figure 5D, *r* = 0.6598, *P* = 0.0196), immobility time on tail suspension test (Figure 5E, *r* = 0.5951, *P* = 0.0412), and cognitive performance in object location task (Figure 5F, *r* = −0.6032, *P* = 0.0379) was found for the poly (I:C) group of animals at 9-week of age. To our surprise, there was no significant difference in the correlation of total dendritic length to different behaviors from the poly (I:C) animals at 12-week of age (Figures 5G–I). Besides, we also did the correlation analyses of the proliferation for postnatal neurogenesis with variety of behavioral indexes from individual poly (I:C) group of animals. We found there was no significant difference in the correlation of DCX positive cells to different behaviors for the poly (I:C) animals at 9-week of age (Figures 5J–L).

Together, these correlation analyses indicated that the delayed emergence of multiple schizophrenia-like symptoms are functionally coupled to the aberrant dendritic elaboration of postnatal neurogenesis instead of the changes of its proliferation in the poly (I:C) group of animals, especially during the critical developmental stage at 9-week of age.

### Increase in the Neurogenesis During the Critical Period of Neurodevelopment Exacerbates the Schizophrenia-Like Pathology

**FIGURE 6:**
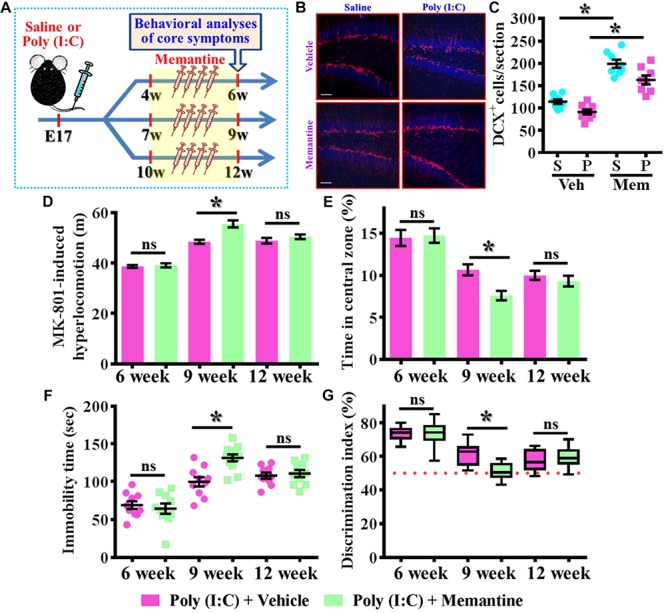
Enhancement of postnatal neurogenesis exacerbates the schizophrenia-like pathology. **(A)** Schematic illustration of the procedure for experimental design. Memantine was injected for a period of 2 weeks before behavioral analyses. **(B)** Representative images for the DCX positive cells from different experimental groups at 9-week-old of age. Red, DCX^+^ cells. Blue, DAPI signal. The scale bar is 50 μm. **(C)** Scatter plots showing the number of DCX positive cells per hippocampal section. Two-way ANOVA: Drug (vehicle vs. MEM): *F*(1,29) = 106.5, *P* < 0.0001; treatment (saline vs. poly I:C): *F*(1,29) = 15.2, *P* = 0.0005; interaction: *F*(1,29) = 0.706, *P* = 0.4076 and analyzed with Bonferroni’s *post hoc* analysis. **(D)** Quantification of MK-801-induced hyperlocomotion with or without memantine treatment. Two-way ANOVA: Age (6, 9, 12): *F*(2,50) = 91.1, *P* < 0.0001; treatment (saline vs. poly I:C): *F*(1,50) = 13.8, *P* = 0.0005; interaction: *F*(2,50) = 6.525, *P* = 0.003. **(E)** Quantification of the percentage in the central open zone. Two-way ANOVA: Age (6, 9, 12): *F*(2,52) = 35.28, *P* < 0.0001; treatment (saline vs. poly I:C): *F*(1,52) = 4.096, *P* = 0.048; interaction: *F*(2,52) = 2.966, *P* = 0.006. **(F)** Scatter plot showing the behavioral performance of tail suspension test. Two-way ANOVA: Age (6, 9, 12): *F*(2,56) = 50.46, *P* < 0.0001; treatment (saline vs. poly I:C): *F*(1,56) = 5.279, *P* = 0.025; interaction: *F*(2,56) = 6.766, *P* = 0.0023. **(G)** Box-and-whisker plots showing the cognitive performance of object location memory task. Two-way ANOVA: Age (6, 9, 12): *F*(2,58) = 48.57, *P* < 0.0001; treatment (saline vs. poly I:C): *F*(1,58) = 3.718, *P* = 0.0587; interaction: *F*(2,58) = 6.523, *P* = 0.0028. Data are presented as mean ± SEM. *n* = 10–12 mice. ^*^P < 0.05. “ns” presents no significant.

To further strengthen the pathological significance of aberrant dendritic development during different time window along the neurodevelopment that contribute to the schizophrenia-like pathology, we next examined if increase the number of these population of aberrant newly generated neurons might exacerbate the pathology of schizophrenia. Experimentally, the intraperitoneally injection of memantine, a well-accepted pharmacological stimulator of neurogenesis was applied for testing the role of postnatal neurogenesis in the pathology of schizophrenia (Figure 6A). We first confirmed that memantine treatment significantly increase in the number of DCX positive cells in the hippocampal dentate gyrus for both saline or poly (I:C) group of animals at 9-week of age (Figures 6B,C, *P* < 0.05 vs. vehicle groups). Then we tested the impact of memantine treatment on different behavioral indexes at 6-, 9-, and 12-week of age. Our data indicated that memantine treatment during the period of seven to 9 week of age for the poly (I:C) group of animals significantly exacerbate the behavioral abnormalities. The animals displayed significant increase in MK-801-induced hyperlocomotion (Figure 6D, 9-week-Vehicle, 48.5 ± 0.7745 m vs. 9-week-memantine, 55.62 ± 1.385 m, *P* < 0.05), decrease in central zone entry (Figure 6E, 9-week-Vehicle, 10.67 ± 0.6522% vs. 9-week-memantine, 7.602 ± 0.5532%, *P* = 0.0321), increase in immobility during tail suspended (Figure 6F, 9-week-Vehicle, 99.7 ± 6.227 s vs. 9-week-memantine, 131.4 ± 4.626 s, *P* < 0.05) and cognitive impairment (Figure 6G, 9-week-Vehicle, 61.69 ± 1.965% vs. 9-week-memantine, 51.04 ± 1.384%, *P* < 0.05). Interestingly, no significant influence of memantine treatment on the schizophrenia related symptoms for the application at either earlier (4- to 6-week old) or later (10- to 12-week) time points which strongly suggested there is a critical time window for the aberrant postnatal neurogenesis that pathophysiologically contributes to the schizophrenia-like pathology.

**FIGURE 7:**
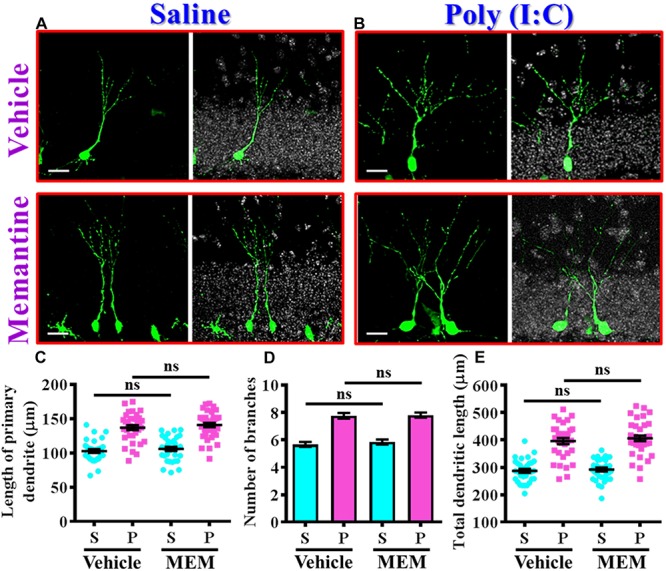
The impact of memantine treatment on the dendritic development of the newly generated neurons. **(A,B)** Representative images for the retroviral labeled newly generated neurons at 14 days post-retroviral injection from saline **(A)** or poly (I:C) **(B)** animals at 9-week-old of age. Green, EGFP signal from labeled cells. White, DAPI signal. The scale bar is 25 μm. **(C)** Scatter plots showing the length of primary dendrite of newly generated neurons with or without memantine treatment. Two-way ANOVA: Drug (vehicle vs. MEM): *F*(1,135) = 1.245, *P* = 0.2665; treatment (saline vs. poly I:C): *F*(1,135) = 124.5, *P* < 0.0001; interaction: *F*(1,135) = 0.0153, *P* = 0.9016. **(D)** Quantification of the number of dendritic branches from newly generated neurons. Two-way ANOVA: Drug (vehicle vs. MEM): *F*(1,135) = 0.3646, *P* = 0.547; treatment (saline vs. poly I:C): *F*(1,135) = 115.1, *P* < 0.0001; interaction: *F*(1,135) = 0.1095, *P* = 0.7412. **(E)** Scatter plots showing the total dendritic length of newly generated neurons. Two-way ANOVA: Drug (vehicle vs. MEM): *F*(1,135) = 0.6339, *P* = 0.4273; treatment (saline vs. poly I:C): *F*(1,135) = 152.8, *P* < 0.0001; interaction: *F*(1,135) = 0.083, *P* = 0.7736. Data are presented as mean ± SEM in **(C–E)**. *n* = 35–40 cells obtained from seven mice. “ns” presents no significant.

Since we found the pharmacological stimulation of neurogenesis by memantine treatment during the critical period of neurodevelopment significantly exacerbates the core symptoms of schizophrenia-like pathology in the poly (I:C) group of animals, we then investigated if the dendritic development of the newly generated neurons might also be affected by memantine treatment. Experimentally, retroviral labeling of newly generating neurons was analyzed at 14 days after retroviral injection for the visualization of dendritic development of postnatal neurogenesis (Figures 7A,B). Similar to our earlier findings, there is an aberrant dendritic development of the newly generated neurons from the poly (I:C) group of animals at 9-week of age, but the treatment of memantine between seven to 9-week age old of the animals has no significant impact on the dendritic development of postnatal neurogenesis (Figures 7A,B). Quantitative analyses indicated that there is no significant difference in the length of primary dendrite (Figure 7C), the number of dendritic branches (Figure 7D) and the total dendritic length (Figure 7E) of the newly generated neurons from either the saline or poly (I:C) group of animals compared between memantine treatment with vehicle treated animals.

Together, these observations indicated that memantine treatment during the critical time window of neurodevelopment significantly increase the number of the population of aberrant newly generated neurons which results in the exacerbation of schizophrenia-like pathology.

### Temporal Suppression of Aberrant Postnatal Neurogenesis Ameliorates the Occurrence of Schizophrenia-Like Pathology

**FIGURE 8:**
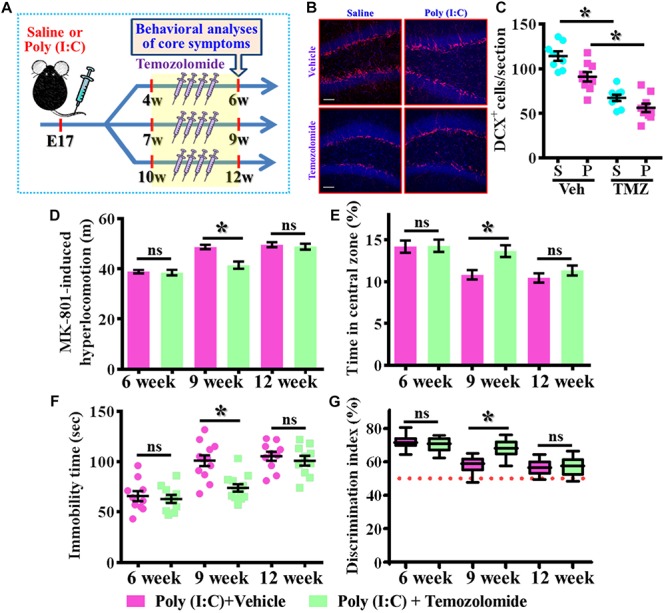
Temporal suppression of aberrant postnatal neurogenesis ameliorates the schizophrenia-like pathology. **(A)** Schematic illustration of the procedure for experimental designs. Temozolomide was injected for a period of 2 weeks before behavioral analyses. **(B)** Representative images for the DCX positive cells from different experimental groups at 9-week-old of age. Red, DCX^+^ cells. Blue, DAPI signal. The scale bar is 50 μm. **(C)** Scatter plots showing the number of DCX positive cells per hippocampal section. Two-way ANOVA: Drug (vehicle vs. TMZ): *F*(1,31) = 72.59, *P* < 0.0001; treatment (saline vs. poly I:C): *F*(1,31) = 12.97, *P* = 0.0011; interaction: *F*(1,31) = 1.523, *P* = 0.2264 and analyzed with Bonferroni’s *post hoc* analysis. **(D)** Quantification of MK-801-induced hyperlocomotion with or without temozolomide treatment. Two-way ANOVA: Age (6, 9, 12): *F*(2,54) = 49.76, *P* < 0.0001; treatment (saline vs. poly I:C): *F*(1,54) = 10.35, *P* = 0.0022; interaction: *F*(2,54) = 6.486, *P* = 0.003. **(E)** Quantification of the percentage in the central open zone. Two-way ANOVA: Age (6, 9, 12): *F*(2,54) = 13.4, *P* < 0.0001; treatment (saline vs. poly I:C): *F*(1,54) = 5.76, *P* = 0.0199; interaction: *F*(2,54) = 2.328, *P* = 0.1072. **(F)** Scatter plot showing the behavioral performance of tail suspension test. Two-way ANOVA: Age (6, 9, 12): *F*(2,58) = 33.71, *P* < 0.0001; treatment (saline vs. poly I:C): *F*(1,58) = 9.269, *P* = 0.0035; interaction: *F*(2,58) = 4.543, *P* = 0.0147. **(G)** Box-and-whisker plots showing the cognitive performance of object location memory task. Two-way ANOVA: Age (6, 9, 12): *F*(2,54) = 37.91, *P* < 0.0001; treatment (saline vs. poly I:C): *F*(1,54) = 4.888, *P* = 0.0313; interaction: *F*(2,54) = 7.289, *P* = 0.0016. Data are presented as mean ± SEM. *n* = 10–12 mice. ^*^P < 0.05. “ns” presents no significant.

Our data supports the idea that presence of aberrant neurogenesis during a critical period of neurodevelopment leads to the pathology of schizophrenia. Next, we wondered if reduction in the aberrant neurogenesis during the critical period of neurodevelopment might, on the other hand, ameliorate the occurrence of schizophrenia-like pathology. Experimentally, the intraperitoneally injection of temozolomide (25 mg/kg), a DNA-alkylating agent that effectively suppressed the active dividing cells including postnatal neurogenesis was applied (Figure 8A). Again, we first confirmed that temozolomide treatment significantly reduced the number of DCX positive cells in the hippocampal dentate gyrus of both saline or poly (I:C) group of animals at 9-week of age (Figures 8B,C, *P* < 0.05 vs. vehicle groups). Then the impact of decrease in neurogenesis on different behavioral indexes was evaluated at 6-, 9-, and 12-week of age. We found temozolomide treatment during the period of seven to 9 week of age for the poly (I:C) group of animals profoundly ameliorated the behavioral abnormalities. Accordingly, the animals displayed significant decrease in MK-801-induced hyperlocomotion (Figure 8D, 9-week-Vehicle, 48.76 ± 0.8776 m vs. 9-week-temozolomide, 41.52 ± 1.427 m, *P* < 0.05), increase in central zone entry (Figure 8E, 9-week-Vehicle, 10.84 ± 0.5574% vs. 9-week-temozolomide, 13.67 ± 0.703%, *P* = 0.0419), reduction in immobility for tail suspension (Figure 8F, 9-week-Vehicle, 100.9 ± 5.315 s vs. 9-week-temozolomide, 73.83 ± 3.661 s, *P* < 0.05) and improve in cognitive performance (Figure 8G, 9-week-Vehicle, 58.08 ± 1.686% vs. 9-week-temozolomide, 68.13 ± 1.77%, *P* < 0.05). No significant influence of temozolomide treatment on the schizophrenia related symptoms for the application at either earlier or later time points, which further strengthen the critical time window for the aberrant neurogenesis in the pathology of schizophrenia.

**FIGURE 9:**
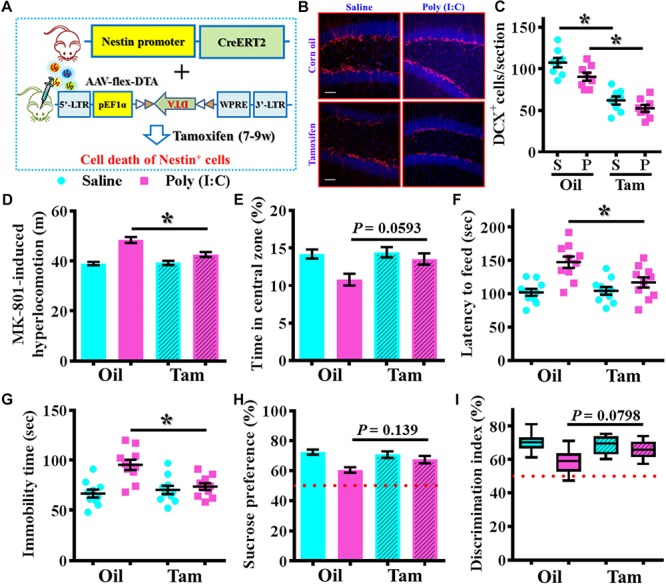
Genetic suppression of aberrant postnatal neurogenesis partially ameliorates the schizophrenia-like pathology. **(A)** Schematic illustration of the procedure for experimental design. Stereotaxic injection of AAV-flex-DTA viruses into the hippocampal SGZ of Nestin-CreERT2 mice and then induce the expression of diphtheria toxin in nestin positive cells by tamoxifen between seven to 9-week of age. **(B)** Representative images for the DCX positive cells from different groups at 9-week-old of age. Red, DCX^+^ cells. Blue, DAPI. Scale bar is 50 μm. **(C)** Scatter plots showing the number of DCX positive cells per hippocampal section. Two-way ANOVA: Genetic induction (oil vs. Tam): *F*(1,28) = 72.32, *P* < 0.0001; treatment (saline vs. poly IC): *F*(1,28) = 7.237, *P* = 0.0119; interaction: *F*(1,28) = 0.5659, *P* = 0.4582 and analyzed with Bonferroni’s *post hoc* analysis. **(D)** Quantification of MK-801-induced hyperlocomotion. Two-way ANOVA: Genetic induction (oil vs. Tam): *F*(1,36) = 8.413, *P* = 0.0063; treatment (saline vs. poly IC): *F*(1,36) = 44.91, *P* < 0.0001; interaction: *F*(1,36) = 10.09, *P* = 0.0031. **(E)** Quantification of the percentage in the central open zone. Two-way ANOVA: Genetic induction (oil vs. Tam): *F*(1,36) = 4.385, *P* = 0.0433; treatment (saline vs. poly IC): *F*(1,36) = 9.13, *P* = 0.0046; interaction: *F*(1,36) = 3.093, *P* = 0.0871. **(F)** Scatter plot showing the latency to feed in the novel context. Two-way ANOVA: Genetic induction (oil vs. Tam): *F*(1,36) = 4.148, *P* = 0.0491; treatment (saline vs. poly IC): *F*(1,36) = 17.42, *P* = 0.0002; interaction: *F*(1,36) = 5.543, *P* = 0.0241. **(G)** Scatter plot showing the behavioral performance of tail suspension test. Two-way ANOVA: Genetic induction (oil vs. Tam): *F*(1,36) = 4.623, *P* = 0.0383; treatment (saline vs. poly IC): *F*(1,36) = 14.29, *P* = 0.0006; interaction: *F*(1,36) = 9.004, *P* = 0.0049. **(H)** Quantification of preference to 3% sucrose solution in the two-bottle preference test. Two-way ANOVA: Genetic induction (oil vs. Tam): *F*(1,36) = 1.621, *P* = 0.2111; treatment (saline vs. poly IC): *F*(1,36) = 13.58, *P* = 0.0007; interaction: *F*(1,36) = 4.315, *P* = 0.045. **(I)** Box-and-whisker plots showing the cognitive performance of object location memory task. Two-way ANOVA: Genetic induction (oil vs. Tam): *F*(1,36) = 2.193, *P* = 0.1473; treatment (saline vs. poly IC): *F*(1,36) = 14.88, *P* = 0.0005; interaction: *F*(1,36) = 4.849, *P* = 0.0342. Data are presented as mean ± SEM. *n* = 10–12 mice. ^*^P < 0.05.

To further confirm the critical period for the aberrant neurogenesis in the development of schizophrenia-like pathology, we also applied a genetic approach to inducible suppress the postnatal neurogenesis in the hippocampal SGZ. Experimentally, we first stereotaxically injected AAV-flex-DTA viruses into the dentate gyrus of Nestin-CreERT2 mice at 5-week-old of the animals. And then, the application of tamoxifen between seven to 9-week of age induced the expression of diphtheria toxin A subunit in Nestin positive cells that results in their targeted cell death (Figure 9A). DCX staining confirmed a significant reduction in the number of DCX positive cells in the dentate gyrus of both saline or poly (I:C) animals at 9-week of age after the injection of tamoxifen (Figures 9B,C, *P* < 0.05 vs. oil injected groups). Behavioral analyses indicated that genetic suppression of aberrant neurogenesis partially ameliorated the schizophrenia-like pathology in poly (I:C) group of animals. As we found the animals displayed significant decrease in MK-801-induced hyperlocomotion (Figure 9D, *P* < 0.05), decrease the latency to feed in the novel context (Figure 9F, *P* < 0.05) and reduction in immobility for tail suspension (Figure 9G, *P* < 0.05). Besides, we also observed a trend increase in central zone entry, sucrose preference and cognitive performance (Figures 9E,H,I) after the genetic suppression of aberrant neurogenesis.

**FIGURE 10:**
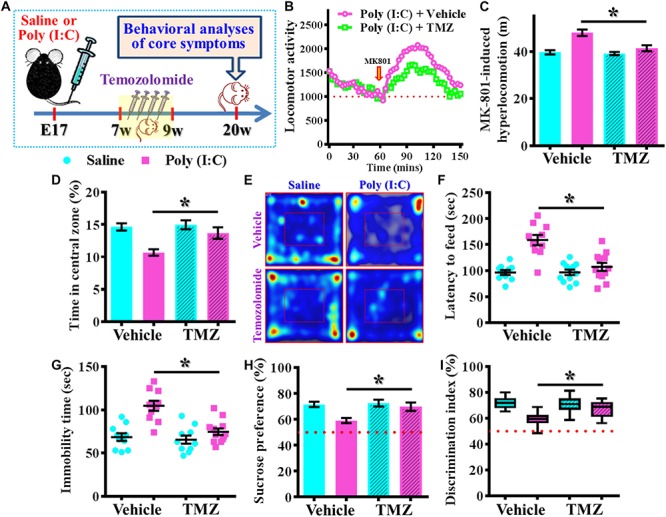
Persisted amelioration of schizophrenia-like pathology by temporal suppression of postnatal neurogenesis during the critical period. **(A)** Schematic illustration of the procedure for experimental designs. Temozolomide was injected between 7- and 9-week of age and behavioral analyses were conducted at their 20-week-old of age for the mice. **(B)** Basal locomotor activity and MK-801-induced hyperlocomotion. Data are presented with the average value from ten mice and each data point represent the cumulative distance traveled for a time bin of 3 min. **(C)** Quantification of MK-801-induced hyperlocomotion. Two-way ANOVA: Drug (vehicle vs. TMZ): *F*(1,36) = 10.65, *P* = 0.0024; treatment (saline vs. poly I:C): *F*(1,36) = 23.36, *P* < 0.0001; interaction: *F*(1,36) = 7.735, *P* = 0.0086 and analyzed with Bonferroni’s *post hoc* analysis. **(D,E)** Quantification and representative images of percentage in the central open zone for the mice. Two-way ANOVA: Drug (vehicle vs. TMZ): *F*(1,36) = 6.06, *P* = 0.0188; treatment (saline vs. poly I:C): *F*(1,36) = 15.01, *P* = 0.0004; interaction: *F*(1,36) = 3.923, *P* = 0.055. **(F)** Scatter plot showing the behavioral performance of novelty suppressed feeding. Two-way ANOVA: Drug (vehicle vs. TMZ): *F*(1,40) = 12.86, *P* = 0.0009; treatment (saline vs. poly I:C): *F*(1,40) = 25.75, *P* < 0.0001; interaction: *F*(1,40) = 13, *P* = 0.0009. **(G)** Scatter plot showing the behavioral performance of tail suspension test. Two-way ANOVA: Drug (vehicle vs. TMZ): *F*(1, 38) = 12.36, *P* = 0.0012; treatment (saline vs. poly I:C): *F*(1, 38) = 23.32, *P* < 0.0001; interaction: *F*(1, 38) = 8.28, *P* = 0.0065. **(H)** Quantification of preference to sucrose solution in two-bottle preference test. Two-way ANOVA: Drug (vehicle vs. TMZ): *F*(1,36) = 5.336, *P* = 0.0267; treatment (saline vs. poly I:C): *F*(1,36) = 9.03, *P* = 0.0048; interaction: *F*(1,36) = 3.668, *P* = 0.063. **(I)** Box-and-whisker plots showing the cognitive performance of object location memory task. Two-way ANOVA: Drug (vehicle vs. TMZ): *F*(1,38) = 3.619, *P* = 0.0647; treatment (saline vs. poly I:C): *F*(1,38) = 18.7, *P* < 0.0001; interaction: *F*(1,38) = 6.379, *P* = 0.0158. Data are presented as mean ± SEM. *n* = 10–12 mice. ^*^P < 0.05.

Next, we wondered if the observed beneficial effect of temporal temozolomide application might be just simply delay the onset of the schizophrenia-pathology or it could feasibly be applied for prevention the emergence of schizophrenia-like pathology in clinic. Experimentally, a short-period suppression of postnatal neurogenesis with temozolomide treatment was applied during the period of seven to 9 week of age for either saline or poly (I:C) group of animals. Then different behavioral indexes that reflect the emergence of schizophrenia-like pathology were evaluated at 20-week of age for the animals (Figure 10A). We found temozolomide treatment during the period of seven to 9 week of age significantly reduced the behavioral abnormalities at their 12-week old of age. Compared to the vehicle treated poly (I:C) animals, temozolomide treatment significantly reduced the MK-801-induced hyperlocomotion [Figures 10B,C, poly (I:C)-Vehicle, 47.97 ± 1.384 m vs. poly (I:C)-temozolomide, 41.37 ± 1.265 m, *P* < 0.05], increased in percentage of central zone entry (Figures 10D,E, poly (I:C)-Vehicle, 10.68 ± 0.502% vs. poly (I:C)-temozolomide, 13.68 ± 0.881%, *P* = 0.0201), decreased the latency to feed in the novel context (Figure 10F, poly (I:C)-Vehicle, 158.7 ± 10.09 s vs. poly (I:C)-temozolomide, 106.9 ± 7.726 s, *P* < 0.05), reduction in immobility for tail suspension (Figure 10G, poly (I:C)-Vehicle, 104.7 ± 5.822 s vs. poly (I:C)-temozolomide, 74.67 ± 3.807 s, *P* < 0.05), elevated preference to sucrose solution (Figure 10H, poly (I:C)-Vehicle, 59.18 ± 1.95% vs. poly (I:C)-temozolomide, 69.88 ± 3.277%, *P* < 0.05) and improve in cognitive performance (Figure 10I, poly (I:C)-Vehicle, 59.15 ± 1.873% vs. poly (I:C)-temozolomide, 67.39 ± 1.886%, *P* < 0.05).

Together, these findings strongly suggested a persisted amelioration of schizophrenia-like pathology by transient/temporal suppression of aberrant neurogenesis during a critical time window along the developmental process.

## Discussion

Postnatal neurogenesis which gives rise to new neurons during the brain maturation through adolescence, adulthood and even later stages providing the substrates for structural and functional remodeling of the neural circuits continuously in the adult brain. Through multiple steps of maturation process including stem cell proliferation, neuronal differentiation, migration, and dendritic elaboration which ultimately integrated into the existing neural circuitry, the postnatal newly generated neurons can actively contribute to critical roles in certain cognitive functions, spatial memory storage and emotional regulation in the adult brain (Aimone et al., 2006; Ming and Song, 2011; Goncalves et al., 2016). Moreover, a continued process of neuronal maturation through the embryonic to the adulthood stages might be particularly vulnerable to variety of genetic or environmental alterations along the brain development. Based on the possible role of abnormal postnatal neurogenesis in the pathology of schizophrenia, targeting of neurogenesis in the adult brain has long been proposed as the potential strategy for the treatment of these neuropsychiatric disorders (Yun et al., 2016). However, direct evidence demonstrating the aberrant postnatal neurogenesis in the pathology of schizophrenia is still missing.

According to the human epidemiological studies for the correlation of prenatal exposure to infection with the higher risk of schizophrenia (Shi et al., 2003), numerous rodent models for schizophrenia have been established to explore the long-term neurodevelopment and behavioral consequences of prenatal immune challenge. Here in our study, the maternal immune activation through the injection of poly (I:C) was applied for the neurodevelopmental model of schizophrenia. Poly (I:C) is recognized by toll-like receptor (TLR) 3 as a viral double-stranded RNA that induces strong innate immune responses (Alexopoulou et al., 2001) which activate multiple events including cytokine-associated inflammation (Meyer et al., 2008), oxidative stress and zinc deficiency (Monte et al., 2017; Khalil, 2018) that pathologically contribute to the emergence of abnormalities relevant to the symptoms of schizophrenia (Richetto et al., 2017).

By using the neurodevelopmental model of schizophrenia of maternal poly (I:C) injection, our current study has provided some valuable information that might display highly clinical implications for the treatment of schizophrenia related pathology through the targeting of aberrant postnatal neurogenesis. At first, the poly (I:C) animals displayed delayed onset of schizophrenia-like pathology along their developmental stages which recapitulate the neurodevelopmental nature of schizophrenic pathology in human patients. Second, we found a phenotypic alteration in postnatal neurogenesis in both the proliferation and dendritic development from poly (I:C) animals along different stages of neurodevelopment. Third, the aberrant dendritic phenotypes instead of the proliferating capacity are pathological correlated with the severity of schizophrenia-like symptoms, especially during the critical developmental stage around 9-week of age for the poly (I:C) animals. Fourth, increase in the neurogenesis during the critical time window between seven to 9-week of age for the poly (I:C) animals exacerbates the schizophrenia-like pathology. And finally, temporal suppression of postnatal neurogenesis during the critical time window resulted in persisted amelioration of the occurrence in schizophrenia-like pathology. These findings provide direct evidence showing the aberrant dendritic development of postnatal neurogenesis during the critical period of brain maturation is essential for the pathophysiological progression of schizophrenia and suggested a feasible approach for temporal manipulation of the postnatal neurogenesis for the amelioration of delayed onset of schizophrenia in clinic.

In our current study, a critical time window for the postnatal neurogenesis that contributes to the development of schizophrenia-like pathology has been confirmed by either increase or decrease in the aberrant neurogenesis between the seven to 9-week-old of age for the poly (I:C) animals. Such developmental period of rodents has long been considered to resemble the late adolescence to early adulthood of human subjects (Dutta and Sengupta, 2016). As the adolescence is a major transitional period from childhood to adulthood, accumulating evidence indicates that the period of adolescence or young adulthood represents an essential stages for the morphological or functional maturation of the neural circuits that processing of higher cognitive performance (Pfefferbaum et al., 1994; Selemon and Zecevic, 2015). Meanwhile, certain functional changes of the brain which including the structural remodeling of the neural circuits associated with emotional regulation and also the cognitive flexibility in processing of executive functions has been reported during the same neurodevelopmental stages (Sisk and Zehr, 2005; Laviola et al., 2009). Meanwhile, the alterations of regional myelination and connectivity between limbic and prefrontal structures have been proposed to underlie the refinement of the efficiency and specificity in information processing (Paus, 2005) along the brain development. Remarkably, accumulating evidence indicates that adolescent period is an extremely vulnerable stage for the central nervous system to be affected by the genetic or environmental stimulus (Holder and Blaustein, 2014). During this critical time window of neurodevelopment, the suffering of environmental disturbance, such as distress, infection or malnutrition might result in maladaptive changes of the brain (Luna et al., 2015) that contribute to the juvenile- or adult-onset of neuropsychiatric disorders in their later developmental stages (Deverman and Patterson, 2009). In our current study, we provide direct evidence showing the functional significance of such time window of the neurodevelopment is critical for the development of schizophrenia-like pathology by the aberrant postnatal neurogenesis.

Postnatal neurogenesis in the adult brain mainly occurs in restricted brain regions, especially the hippocampal dentate gyrus. The hippocampus has long been associated with the function of emotional regulation and cognitive performance (Sapolsky, 2000) and accumulating evidence showing the dysfunction of hippocampal formation is one of the endophenotypes of schizophrenia (Walton et al., 2012; Hagihara et al., 2013). Besides, previous rodent model of neonatal ventral hippocampal lesion results in adolescent-onset of schizophrenic-like behaviors in rats (Lipska et al., 2002; Sandner et al., 2014) further strengthens our findings that aberrant postnatal neurogenesis in the hippocampal SGZ during the critical period of brain maturation pathologically contributes to the delayed emergence of schizophrenia-like pathology.

For the critical time window of aberrant neurogenesis in the formation of schizophrenia pathology, we found only the manipulation between seven to 9-week-old of age for the animals, either increase or decrease the population of aberrant neurogenesis affect the schizophrenia-like symptoms. The lack of effect for later time point cannot be simply explained by the ceiling effect of behavioral analyses. By testing the positive symptoms with a lower dose of MK-801 (0.2 mg/kg), the increase in neurogenesis by memantine treatment between 10–12 week of age still does not change the schizophrenia-like pathology significantly. Besides, loss of function experiments with temozolomide only ameliorated the behavioral abnormalities if applied during the period of 7–9 week of age but not the later stages further strengthen that such developmental stages as the critical time window for aberrant postnatal neurogenesis that contributes to the onset of schizophrenia-like pathology later.

Meanwhile, compared to the significant amelioration of schizophrenia-like pathology by temozolomide treatment during the critical time window, the genetic approach with diphtheria toxin expression in Nestin positive cells only partially reversed the schizophrenia-like behaviors in the poly (I:C) animals. Few possibilities might contribute to such discrepancy between the pharmacological and genetic experiments. First, though both approach displayed similar efficacy in the reduction of DCX positive cells after 2 week of treatment, the delayed onset in its effect with genetic manipulation might miss the early stage of the critical time window. Second, the local injection of viruses in the dentate gyrus by genetic manipulation only suppress neurogenesis in the hippocampus which the postnatal neurogenesis happened in other neurogenic regions such as subventricular zone might display certain roles in the development of schizophrenia-like behaviors. Third, the non-specific suppression in the proliferation of other cell types in the brain such as microglia or endothelial cells by temozolomide might cause regional inflammation that altered animal behaviors or neurogenesis indirectly. Further detail experiments might be required in the future to answer these possibilities.

In conclusion, through comprehensive behavioral analyses at different developmental stages of the poly (I:C) challenged offspring, we observed a delayed onset of schizophrenia-like pathology and the severity of the symptoms positively correlated with the aberrant dendritic phenotypes preferentially at 9-week-old of age for the animals. Temporal suppression of aberrant neurogenesis during such critical time period ameliorates the emergence of schizophrenia-like symptoms. These findings provide solid evidence showing the role of aberrant postnatal neurogenesis in the pathology of schizophrenia and suggest a feasible therapeutic strategy for the treatment of schizophrenia by targeting of the aberrant neurogenesis through the critical time window of neurodevelopment.

## Data Availability

The raw data supporting the conclusions of this manuscript will be made available by the authors, without undue reservation, to any qualified researcher.

## Ethics Statement

All animal experiments and care procedures conformed to the Guide for the Care and Use of Laboratory Animals (LAC-2017-0174) and were approved by the Institutional Animal Care and Use Committee of Taipei Medical University.

## Author Contributions

J-RS, C-YH, and C-HY conceived and designed the experiments. C-YH, TJ, M-FT, H-NL, and S-WH performed the experiments and analyzed the data. TJ and MM contributed toward editing the manuscript. J-RS and C-HY wrote the manuscript. All authors discussed, edited, and approved the final version of the manuscript.

## Conflict of Interest Statement

The authors declare that the research was conducted in the absence of any commercial or financial relationships that could be construed as a potential conflict of interest.
